# 61. An unusual case of blue digits

**DOI:** 10.1093/rap/rky034.024

**Published:** 2018-09-20

**Authors:** David J T McCormick, Mark Leith, Roland McKane, Anita Smyth

**Affiliations:** Rheumatology, Ulster Hospital, Dundonald, Belfast, UNITED KINGDOM

**Introduction:** This is an interesting case regarding a lady that initially presented with painful discoloured digits. At first they appeared as raynauds or acrocyanosis effecting the 2nd, 3rd, 4th, 5th digits of both hand with sparing of the thumbs. There were associated with high inflammatory markers and fevers. Workup for infection and malignancy were negative. Digital ischaemia progressed to ultimately become digitial necrosis of finger tips. Autoimmune screens were initially normal, however, anti-Ro antibody subsequently became strongly positive and anti-PL12 also later returned positive in keeping with a diagnosis of anti-synthetase syndrome. In addition to this, the onset of dyspnoea resulted in high resolution CT of chest showing cryptogenic organising pneumonia with climbing oxygen demands. After an initial response to oral steroid, therapy was escalated to intravenous methylprednisolone and cyclophosphamide with improvement in respiratory function as well as imaging and halting of digital necrosis. We await the outcome of ongoing immunosuppressive therapy.

**Case description:** This 77 year old lady with a background of ischaemic heart disease and previous cardiac bypass presented to hospital in March 2018 with discomfort in her finger tips and a blue discolouration in keeping with raynauds or acrocyanosis. There had been a preceding lower respiratory tract infection. Investigations were underway to assess for any infective or malignant aetiology with negative cultures, viral screen, echocardiogram and ct scan of chest, abdomen and pelvis. Initial autoimmune screen showed a weakly positive anti-Ro antibody and equivocal lupus anticoagulant. Ultrasound Doppler of hand revealed echolucent thrombus within the proper digital arteries bilaterally effecting the 2nd, 3rd, 4th and 5th digits and sparing of the princeps pollicis artery to the thumbs. Vascular supply proximal to these areas was normal on Doppler. Fevers were intermittent during this early phase of hospital admission with raised inflammatory markers. Antibiotics were ineffective and therefore a trial of prednisolone 40mg daily was prescribed with settling of fevers and crp within 48 hours. Unfortunately the digital ischaemia progressed to digital necrosis of the finger tips and progressive shortness of breath lead to high resolution ct of chest showing cryptogenic organising pneumonia which had not been on imaging one month prior. Intravenous iloprost in addition to calcium channel blockers and therapeutic doses of enoxaparin had not made any clinical improvement. Repeat autoimmune screen returned anti-Ro antibody now strongly positive and with digital necrosis, HRCT findings, climbing oxygen demands, steroid responsiveness it was felt an underlying connective tissue disease was the most likely aetiology. Therapy was escalated to intravenous methylprednisolone 750mg for three pulses and cyclophosphamide 10mg/kg followed by prednisolone 60mg daily. A myositis panel had been sent and returned after administration of the first dose of cyclophosphamide revealing a positive anti- PL12 antibody. This alongside the digital necrosis and interstitial lung disease are in keeping with a diagnosis of antisynthetase syndrome. Cyclophosphamide pulses are ongoing with weaning doses of prednisolone alongside sildenafil. The digital ischaemia has halted and the necrotic lesions have become well demarcated and beneath the necrotic tissue, healthy cutaneous tissue is slowly being revealed. Chest x-ray has dramatically improved and oxygen demands have reduced to as required use of 1-2 litres via nasal cannula. Repeat ultrasound Doppler of hands shows echolucent thrombus persisting within the proper digital arteries. It does, however, show development of collateral vessels around the occlusions. Treatment plans are to complete the course of pulsed cyclophosphamide and wean steroid dose down accordingly. Reducing oxygen demands are encouraging with regards this lady's chest disease and we will await the overall outcome and hope progress continues to be made.

**Discussion:** Antisynthetase syndrome is a rare condition which is a variant of polymyositis/dermatomyositis. It is an idiopathic inflammatory myopathy. It appears to have a higher prevalence for interstitial lung disease than polymyositis or dermatomyositis. Often there is no or subclinical myositis. Digital ischaemia and necrosis is described in case reports alongside interstitial lung disease in those who are positive for anti-PL12 antibody. Anti- Ro antibody also appears to be commonly positive in this group of patients. Due to its rarity there are no guidelines with regard to treatments. Evidence for therapeutic intervention is based on case reports with glucocorticoid as the mainstay of acute treatment. Several DMARD therapies have been described as having benefit. Rituximab and cyclophosphamide have been used a rescue therapies in case reports and that is what was felt to be required in this case.

**Key Learning Points:** Evolving connective tissue diseases can prove difficult to diagnose. It is important to exclude other differentials especially in the case of infective aetiologies prior to any immunosuppressive therapy initiation. Requesting the myositis panel is what clinched the diagnosis in this case. Treatment, however, was already underway based on the clinical findings and positive anti-Ro antibody.

**Disclosure: D.J.T. McCormick:** None. **M. Leith:** None. **R. McKane:** None. **A. Smyth:** None.

